# Identification and analysis of MSC-Exo-derived LncRNAs related to the regulation of EMT in hypospadias

**DOI:** 10.1186/s12920-024-01869-9

**Published:** 2024-04-16

**Authors:** Mengmeng Chang, Hongjie Gao, Yingying Li, Chen Ding, Zhiyi Lu, Ding Li, Fan Huang, Jiawei Chen, Fengyin Sun

**Affiliations:** 1 Department of Pediatric Surgery, Qilu Hospital of Shandong University, Jinan, China; 2 Department of Pediatrics, Qilu Hospital of Shandong University, Jinan, China

**Keywords:** Hypospadias, Bioinformatics analysis, EMT, MSC-Exos, HCG18

## Abstract

**Objective:**

This study aims to screen the differentially expressed long non-coding RNAs (DELncRNAs) related to the regulation of epithelial-mesenchymal transition (EMT) in hypospadias in mesenchymal stem cell-derived exosomes (MSC-Exons) and explore the potential mechanism of these lncRNAs for the EMT in hypospadias.

**Methods:**

In this study, the microarray data related to MSC-Exos and hypospadias were downloaded from Gene Expression Omnibus (GEO). Besides, the lncRNAs highly expressed in MSC-Exos and the differentially expressed mRNAs and lncRNAs in children with hypospadias were screened, respectively. In addition, the lncRNAs enriched in MSC-Exos and differentially expressed lncRNAs in hypospadias were intersected to obtain the final DElncRNAs. Moreover, the co-expression interaction pairs of differentially expressed lncRNAs and mRNAs were analyzed to construct a Competing Endogenous RNA (ceRNA) network. Finally, the candidate lncRNAs in exosomes were subjected to in vitro cell function verification.

**Results:**

In this study, a total of 4 lncRNAs were obtained from the microarray data analysis. Further, a ceRNA regulatory network of MSC-Exo-derived lncRNAs related to the regulation of EMT in hypospadias was constructed, including 4 lncRNAs, 2 mRNAs, and 6 miRNAs. The cell function verification results indicated that the exosomes secreted by MSCs may transport HLA complex group 18 (HCG18) into target cells, which promoted the proliferation, migration, and EMT of these cells.

**Conclusion:**

MSC-Exo-derived lncRNA HCG18 can enter target cells, and it may be involved in the regulation of EMT in hypospadias through the ceRNA network.

**Supplementary Information:**

The online version contains supplementary material available at 10.1186/s12920-024-01869-9.

## Introduction

Hypospadias is one of the most common congenital malformations in male children, and this condition is caused by abnormal urethral development [1, 2]. Surgery is still the only therapeutic method for hypospadias in clinical practice. However, some serious complications, such as urinary fistula and urethral stricture, may be induced after surgical treatment. This can be mainly attributed to the poor regeneration and healing ability of urethral tissues [3]. Therefore, there is an urgent demand for clarifying the pathogenic mechanism of hypospadias to promote wound healing and improve surgical outcomes [4]. Epithelial-mesenchymal transition (EMT) is a basic biological process [5]. Based on that, epithelial cells can be transformed into mesenchymal cells to generate or regenerate tissues. Transforming growth factor-β (TGF-β) and fibroblast growth factor (FGF) are the main growth factors related to urethral development [6, 7]. Currently, these growth factors are verified to correlate closely with EMT. Additionally, it has been demonstrated that EMT dysregulation is vital for the pathogenesis of hypospadias, but the specific mechanism needs further exploration [8–10].

Mesenchymal stem cells (MSCs) are a group of stem cells with self-renewal and multidirectional differentiation potential. Mesenchymal stem cell-derived exosomes (MSC-Exos) contain specific nucleic acids and proteins, which can exchange genetic information between cells [11]. As revealed in multiple studies, MSC-Exos play a crucial role in the regulation of EMT in many diseases through its paracrine. For example, Grange et al. confirmed that miR-294 and miR-133 in MSC-Exos can improve the EMT of the renal tubular by inhibiting Smad2 phosphorylation induced by TGF-β1, thus delaying renal interstitial fibrosis [12]. Yao et al. found that MSC-Exos inhibited the EMT process of endometrial epithelial cells via the TGF-β1/Smad signaling pathway, thus promoting endometrial repair [13]. Xiao et al. reported that miR-23a-3p and miR-182-5p transferred by MSC-Exos inhibited the NF-κB and Hedgehog pathways by silencing Ikbkb and destabilizing IKKβ, thus reversing the EMT process in LPS-induced pulmonary injury [14].

Meanwhile, MSC-Exos promoted the in vitro proliferation and migration of urethral smooth muscle cells (USMCs) in children with hypospadias by activating the CD73/adenosine signaling axis and downstream PI3K/AKT pathway, which may promote urethral tissue regeneration and repair [15]. However, it remains unclear whether MSC-Exos can improve urethral regeneration and repair by promoting EMT in hypospadias. In this study, the MSC-Exo-derived lncRNAs related to the regulation of EMT in hypospadias were screened based on the data from GEO to construct the lncRNA-miRNA-mRNA network. Besides, new therapeutic targets for hypospadias tissue repair were also identified. These findings are expected to reduce the incidence of such complications as urinary fistula and urethral stricture after the surgical treatment of hypospadias.

## Materials and methods

### Data acquisition, data preprocessing, and lncRNA re-annotation

In this study, the gene expression profile of exosomes derived from primary MSCs isolated from human bone marrow tissues (GSE12243, GPL6102) and microarray expression data and clinical information associated with hypospadias (GSE35034, GPL14550) were downloaded from Gene Expression Omnibus (GEO, https://www.ncbi.nlm.nih.gov/gds).

ID conversion was performed via the R package "org.Hs.eg.db", and Perl was employed to merge these sample data and re-annotate these genes. In addition, the probe codes were matched with corresponding mRNAs/lncRNAs (gene symbols) to remove the unmatched probes. When different probes were mapped to the same gene, the mean value of these probes was calculated as the final expression level of the gene. The top 100 most highly expressed lncRNAs were screened by ranking the gene expression of MSC-derived exosomes.

### Screening of differentially expressed genes and lncRNAs

The differential expression analysis of all mRNAs and lncRNAs encoding protein was performed by the "limma" package of R based on the screening criteria of *P* < 0.05 and |logFC|> 0.5. Subsequently, the heatmap and the volcano plot of differentially expressed mRNAs/lncRNAs were drawn by the "pheatmap" and "ggplot2" packages.

The top 100 MSC-Exo-derived lncRNAs with the highest expression were selected, and the differentially expressed mRNAs and lncRNAs in children with hypospadias were screened. Finally, the lncRNAs with the highest expression in MSC-Exos and differentially expressed lncRNAs in hypospadias were intersected to obtain the final DElncRNAs with the assistance of the "venn" package of R.

### Screening of EMT-related DElncRNAs

EMT-related genes were collected from the HALLMARK_EPITHELIAL_MESENCHYMAL_TRANSITION gene set in Molecular Signatures Database (MSigDB, v7.1, https://www.gsea-msigdb.org/gsea/msigdb/). Through Pearson correlation analysis, EMT-related lncRNAs were identified according to *P* < 0.05 and the absolute Pearson coefficient > 0.6.

### Prediction of the co-expression interaction between EMT-related DElncRNAs and DEmRNAs

The Pearson correlation coefficients of differentially expressed mRNAs and EMT-related DElncRNAs were calculated based on the mRNA and lncRNA data of matched samples. Besides, correlation tests were performed to screen the interaction pairs that conformed to *r* > 0.6 and *P* < 0.05. Specifically, through the R software Correlation analysis was accomplished by using the function cor () to calculate the correlation coefficient; cor.test () to test the correlation between paired samples, and return both the correlation coefficient and the significance level of the correlation (*p*-value). See Supplementary file 2 for specific code.

### Prediction of miRNAs and construction of a ceRNA network

For co-expressed DElncRNAs, the targeted miRNA prediction was performed with the aid of two tools. Specifically, miRCode/ miRanDa (v3.3a) was employed to perform the prediction based on default parameters. The miRNA-lncRNA interaction pairs were screened as per Score ≥ 140 and Energy ≤ -20. Subsequently, the lncRNA-miRNA interaction pairs were predicted by an online database starBase v2.0. The intersection of the prediction results in 2 batches was taken as the final lncRNA-miRNA interaction pairs. For the mRNAs co-expressed with DElncRNAs, those appearing in the prediction results based on miRWalk, miRTarBase, miRDB, and Targetscan were identified as the miRNAs that can regulate the target gene [16–19]. The miRNA-lncRNA-mRNA interaction pairs regulated by the same miRNA were screened based on differentially expressed lncRNA-miRNA and mRNA-miRNA interaction pairs with differential expression. Then, these interaction pairs were further screened. Next, the ceRNA network was constructed with Cytoscape. The co-expressed lncRNAs and mRNAs regulated by the same miRNA in the ceRNA network can be considered to be a pair of ceRNAs.

### Enrichment analysis of differentially expressed genes related to lncRNAs

The clusterProfiler package in R was utilized to perform the Gene Ontology (GO) and Kyoto Encyclopedia of Genes and Genomes (KEGG) enrichment analyses on the differentially expressed genes (DEGs) related to lncRNAs, with the adjusted *P* < 0.05 as the threshold. Then, the top 10 results of biological process (BP), cellular component (CC), and molecular function (MF) in GO and KEGG enrichment analyses were obtained with the aid of "ggplot2" and "GOplot" packages in R.

### Screening and verification of candidate lncRNAs

Human bone marrow-derived mesenchymal stem cells (MSCs) were purchased from Sciencell (batch No.: 7530). The primary foreskin fibroblasts from children with hypospadias were used as target cells. Exosomes are small vesicles that cells secrete to transmit information between cells. They contain proteins, RNA, lipids, and other biomolecules. GW4869 is a widely used inhibitor of exosome production. It works by inhibiting ceramide synthase (neutral sphingomyelinase (nSMase)), which blocks ceramide-dependent exosome production. The study conducted experiments on two groups: the Co-culture of MSCs and target cells (Co-MSC) group and the MSCs with target cells + exosome inhibitor GW4869 (Co-MSCs + GW4869) group. The culture chambers (PET membrane, 24 mm, pore size 0.4 um) were placed on 6-well plates. MSCs were added to the upper layer and fibroblasts to the lower layer. After evenly distributing the two types of cells in each layer for 48 h, we used a CCK8 assay to detect the effect of the co-culture system on target cell proliferation. We also used Transwell and cell scratch assays to detect changes in target cell proliferation and migration. Additionally, we detected the expression of EMT markers (E-cadherin and N-cadherin) using Western blot. After co-culture in each experimental group, the transcriptional inhibitor 5,6-dichloro-1-β-D-ribofuranosylbenzimidazole (DRB), a commonly used transcriptional inhibitor, was used to inhibit the synthesis of endogenous HCG18. The target cells were treated with the transcriptional inhibitor DRB (350 mg/L) for 1 h. The expression level of exosome-derived HCG18 was then indirectly demonstrated using a Real-time quantitative PCR (RT-qPCR) assay.

### Statistical methods

All the data in this study were analyzed by R, and the difference was analyzed by the Bayes' theorem. Besides, the correlation was analyzed by Pearson correlation analysis. The data between the two groups were analyzed by the two-tailed t test or the Welch t test when appropriate. *P* < 0.05 indicated that the difference was statistically significant.

## Results

### Basic information of differentially expressed genes

**Fig. 1:**
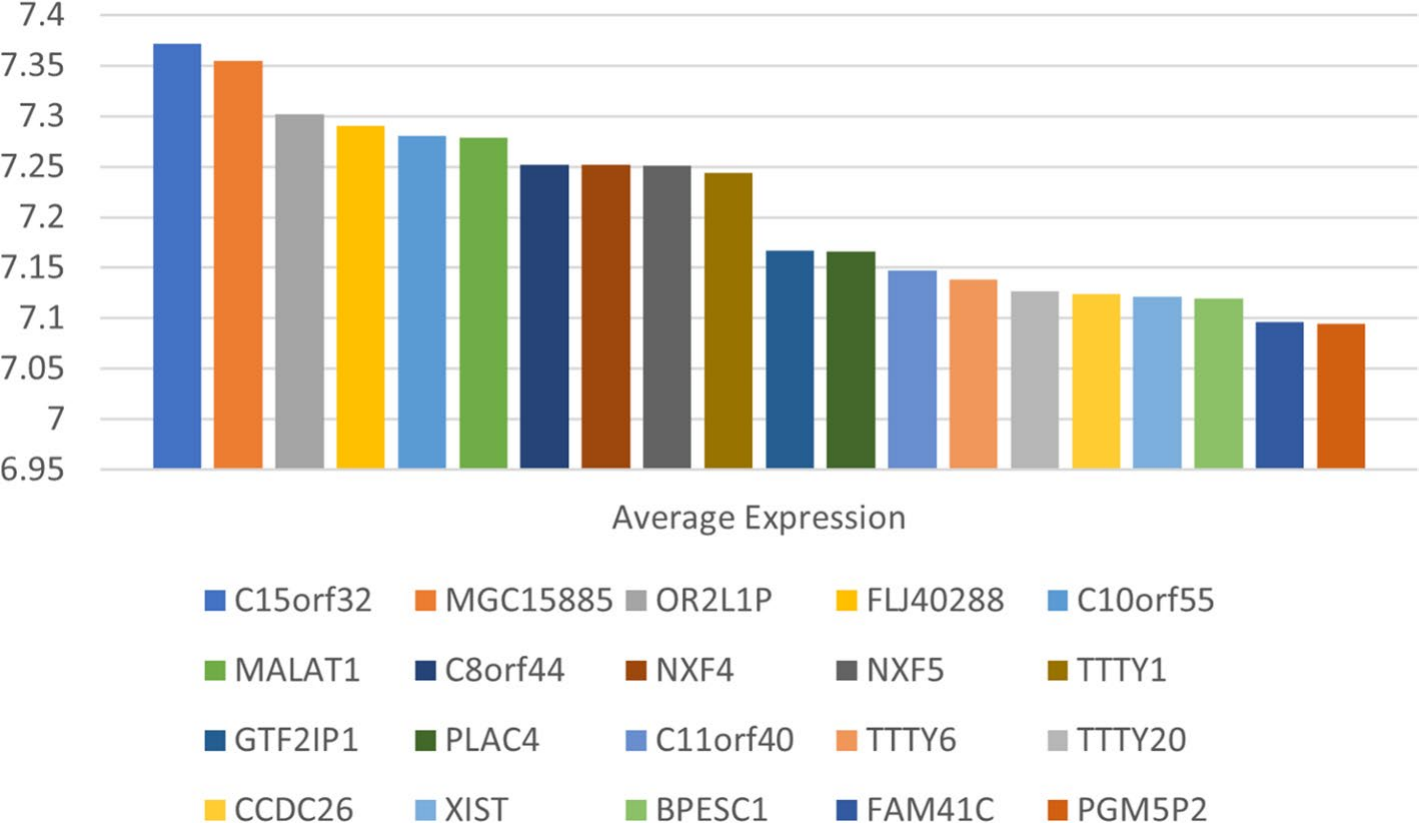
The top 20 lncRNAs with the highest expression in MSC-Exos

**Fig. 2:**
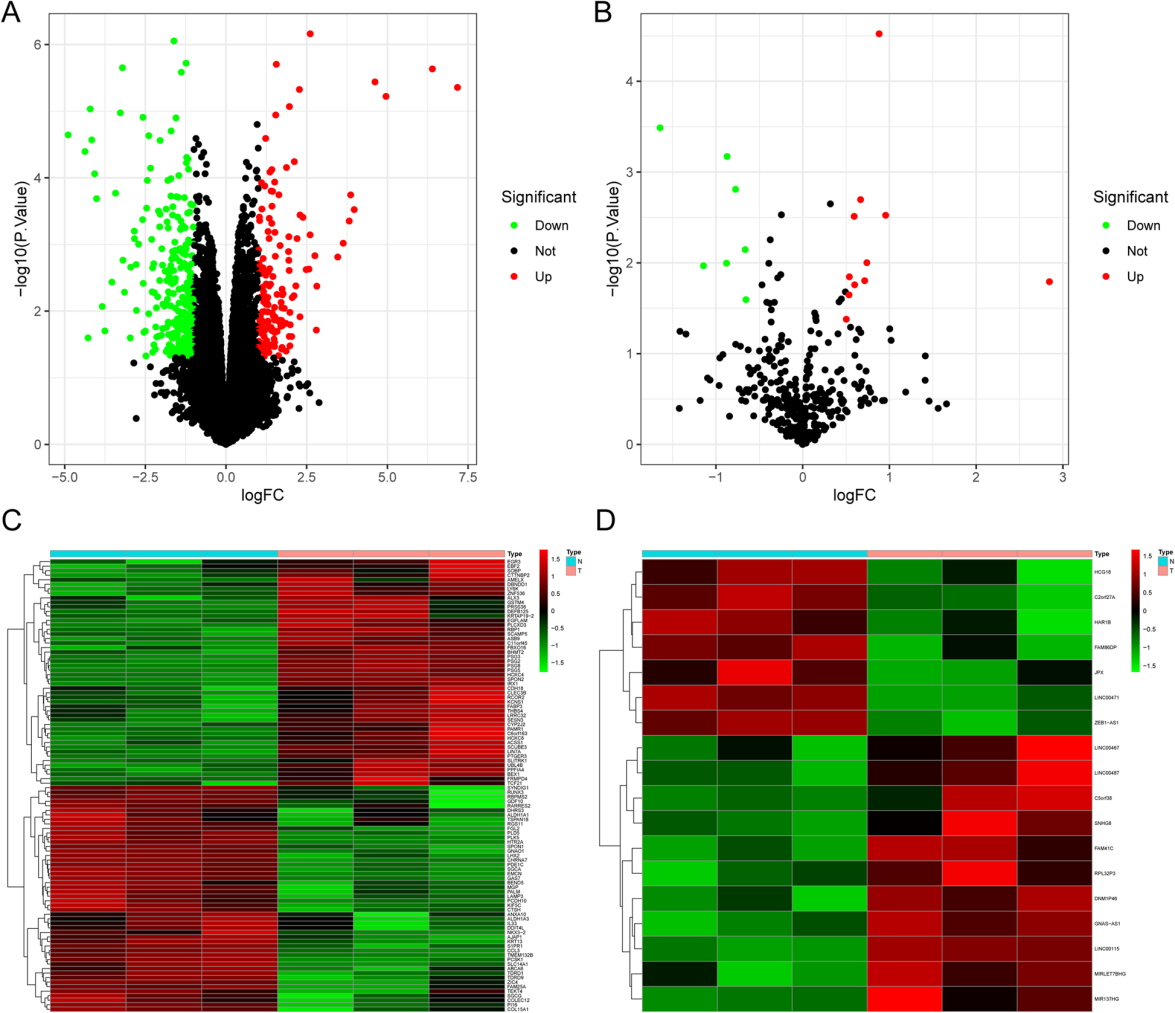
Volcano plots and heatmaps of differentially expressed mRNAs and lncRNAs: In volcano plots **A** and **B**, green represents the low expression and red represents the high expression. In heatmaps **C** and **D**, blue represents the control group, and red represents the hypospadias group

In this study, about 106 lncRNAs were identified in MSC-Exos. The top 20 lncRNAs with the highest expression are shown in Fig. 1. The differential expression analysis of all mRNAs and lncRNAs related to hypospadias was performed by the "limma" package. Eventually, 433 differentially expressed mRNAs and 18 differentially expressed lncRNAs were obtained (Fig. 2). The lncRNAs highly expressed in MSC-Exos and differentially expressed lncRNAs in hypospadias were intersected. Based on that, 4 differentially expressed lncRNAs were obtained, including 1 down-regulated lncRNA and 3 up-regulated lncRNAs. They were ranked based on the *P*-value, and the screened lncRNAs are listed in Table 1.

**Table 1:** The final screening lncRNAs

lncRNA	log FC	*P*
**FAM41C**	0.593372	0.003074
**HCG18**	-0.87905	0.010085
**SNHG8**	0.714734	0.015725
**RPL32P3**	0.599008	0.017453

### Identification of EMT-related DElncRNAs

A total of 200 EMT-related genes were identified from MSigDB. Then, these EMT-related DElncRNAs were screened by correlation analysis. Subsequently, 102 pairs of EMT-related DElncRNAs were identified based on *P* < 0.05 and the absolute Pearson coefficient > 0.6. The top 10 interaction pairs ranked based on their correlation coefficients are listed in Table 2.

**Table 2:** Correlation analysis of EMT-DElncRNA

EMTmRNA	DElncRNA	cor	*p* value	Regulation
**INHBA**	HCG18	0.91727302	2.65E-05	postive
**SPOCK1**	HCG18	0.916286479	2.81E-05	postive
**CRLF1**	HCG18	0.911297517	3.72E-05	postive
**FSTL3**	HCG18	0.901228948	6.26E-05	postive
**SGCB**	SNHG8	-0.821581427	0.001046502	negative
**SERPINE1**	RPL32P3	-0.836083005	0.00070288	negative
**SLIT2**	RPL32P3	-0.843043961	0.000572815	negative
**DPYSL3**	HCG18	-0.882911066	0.000141951	negative
**GLIPR1**	HCG18	-0.884128302	0.00013501	negative
**PLOD3**	HCG18	-0.890180985	0.000104339	negative

### Prediction of co-expression interactions between lncRNAs and mRNAs

A total of 384 co-expressed mRNA-lncRNA pairs were screened, including 330 mRNAs and 4 lncRNAs. The top 15 interaction pairs ranked based on their correlation coefficients are listed in Table 3.

**Table 3:** Correlation analysis of mRNA-lncRNA pairs

DEmRNA	DElncRNA	cor	*p* value	Regulation
**BARHL1**	FAM41C	-0.735556816	0.006401345	negative
**LGI4**	FAM41C	0.673505318	0.01634889	postive
**ANKRD53**	FAM41C	0.621982403	0.030813133	postive
**KRT83**	HCG18	0.980031275	2.42E-08	postive
**CADM1**	HCG18	0.970733334	1.61E-07	postive
**RARRES2**	HCG18	0.963544577	4.77E-07	postive
**PAMR1**	HCG18	-0.965160132	3.81E-07	negative
**C5orf58**	HCG18	-0.968262717	2.40E-07	negative
**MRAP2**	HCG18	-0.97452483	8.10E-08	negative
**MCTP2**	RPL32P3	0.873951848	0.000202043	postive
**MAF**	RPL32P3	0.781446987	0.002684881	postive
**ATAD2**	RPL32P3	-0.851856853	0.000435814	negative
**FABP3**	SNHG8	0.805949572	0.001548615	postive
**NR1D1**	SNHG8	-0.808964271	0.001439781	negative
**DCLRE1B**	SNHG8	-0.837584938	0.000673056	negative

### Prediction of miRNAs and construction of a ceRNA network

**Fig. 3:**
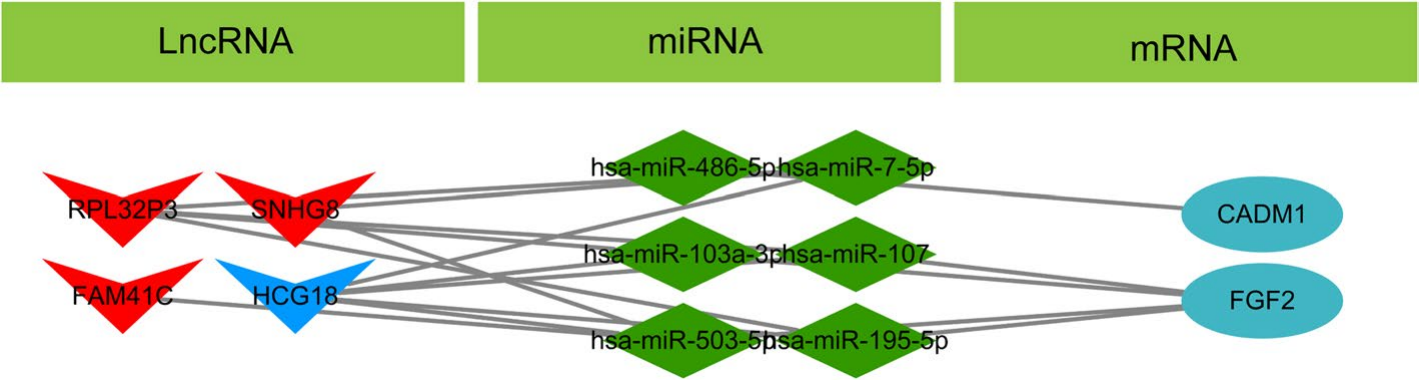
Establishment of a ceRNA network. lncRNAs, miRNAs, and mRNAs are represented by arrows, diamonds, and ellipses, respectively. Red nodes represent the high expression, and blue nodes represent the low expression

The prediction results based on miRCode and starBase revealed that 4 co-expressed DElncRNAs interacted with 63 miRNAs (Table 4). Subsequently, 5 miRNAs targeted by mRNAs co-expressed with DElncRNAs were predicted based on miRWalk, miRTarBase, miRDB, and Targetscan (Table 5). Then, the miRNA-lncRNA-mRNA interaction pairs regulated by the same miRNAs were screened, and the ceRNA network was constructed by combining the co-expression interaction between mRNAs and lncRNAs (correlation coefficient > 0.6). The ceRNA network is shown in Fig. 3. This network comprised 34 sets of miRNA-lncRNA-mRNA regulatory relationships, including 6 miRNAs, 4 lncRNAs, and 2 mRNAs.

**Table 4:** Representative relationships between IncRNAs and miRNAs–63

lncRNA	miRNA				
**HCG18**	hsa-miR-7-5p	hsa-miR-152-3p	hsa-miR-148b-3p	hsa-miR-32-5P	hsa-miR-454-3p
	hsa-miR-9-5P	hsa-miR-153-3p	hsa-miR-181a-5p	hsa-miR-93-5P	hsa-miR-497-5p
	hsa-miR-16-5P	hsa-miR-191-5p	hsa-miR-181b-5p	hsa-miR-96-5P	hsa-miR-503-5p
	hsa-miR-17-5P	hsa-miR-195-5p	hsa-miR-181c-5p	hsa-miR-101-3P	hsa-miR-103-3p
	hsa-miR-24-3P	hsa-miR-205-5p	hsa-miR-181d-5p	hsa-miR-107-5p	hsa-miR-106a-5p
	hsa-miR-25-3P	hsa-miR-218-5p	hsa-miR-301a-3p	hsa-miR-122-5P	hsa-miR-200a-5p
	hsa-miR-31-5P	hsa-miR-424-5p	hsa-miR-302a-3p	hsa-miR-124-3P	hsa-miR-33a-5p
	hsa-miR-302c-3p	hsa-miR-132-3P	hsa-miR-30d-3p	hsa-miR-365b-3p	hsa-miR-106b-5p
	hsa-miR-30a-3p	hsa-miR-138-5P	hsa-miR-33b-3p	hsa-miR-449b-5p	hsa-miR-125b-5p
	hsa-miR-30b-3p	hsa-miR-139-5P	hsa-miR-34a-5p	hsa-miR-520c-5p	hsa-miR-148a-3p
	hsa-miR-30c-5p	hsa-miR-365a-3p			
**RPL32P3**	hsa-miR-7-5p	hsa-miR-16-5P	hsa-miR-24-3P	hsa-miR-124-3P	hsa-miR-138-5P
	hsa-miR-139-5P	hsa-miR-145-5p	hsa-miR-195-5p	hsa-miR-218-5p	hsa-miR-424-5p
	hsa-miR-103-3p	hsa-miR-33a-5p	hsa-miR-34a-5p		
**SNHG8**	hsa-miR-7-5p	hsa-miR-141-5p	hsa-miR-503-5p	hsa-miR-148a-3p	hsa-miR-30c-5p
	hsa-miR-16-5P	hsa-miR-152-3p	hsa-miR-103-3p	hsa-miR-148b-3p	hsa-miR-30e-5p
	hsa-miR-17-5P	hsa-miR-183-5p	hsa-miR-33a-5p	hsa-miR-181a-5p	hsa-miR-34a-5p
	hsa-miR-24-3P	hsa-miR-191-5p	hsa-miR-10a-5p	hsa-miR-301a-3p	hsa-miR-454-3p
	hsa-miR-25-3P	hsa-miR-218-5p	hsa-miR-125b-5p	hsa-miR-30a-3p	hsa-miR-455-5p
	hsa-miR-124-3P	hsa-miR-139-5P			
**FAM41C**	hsa-miR-101-3P	hsa-miR-124-3P	hsa-miR-139-5P	hsa-miR-141-5p	hsa-miR-145-5p
	hsa-miR-183-5p	hsa-miR-205-5p	hsa-miR-455-5p	hsa-miR-503-5p

**Table 5:** Representative relationships between mRNAs and miRNAs

miRNA	RefseqID	mRNA
**hsa-miR-486-5p**	NM_001301043	CADM1
**hsa-miR-103a-3p**	NM_002006	FGF2
**hsa-miR-107**	NM_002006	FGF2
**hsa-miR-195-5p**	NM_002006	FGF2
**hsa-miR-503-5p**	NM_002006	FGF2

### GO and KEGG enrichment analyses

**Fig. 4:**
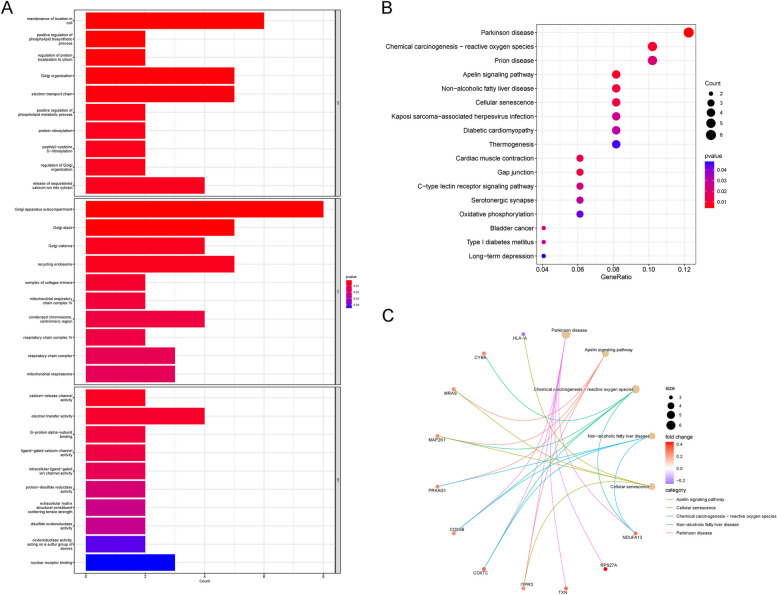
**A** Histogram of GO enrichment analysis. The change of colors correlates with the *P* value, and the column length is positively correlated with the number of associated genes. **B** Bubble chart and **C** chord diagram of KEGG enrichment pathways. The bubble size represents the number of enriched genes, and the color represents the enrichment correlation

The GO enrichment results demonstrated that these DEGs were mainly enriched in biological processes (BPs), cellular components (CCs), and molecular functions (MFs). In terms of BPs, they were mainly enriched in cell repair, positive regulation of phospholipid biosynthesis, protein regulation, and Golgi apparatus regulation. In terms of CCs, they were mainly enriched in Golgi cisternae, mitochondria, and respiratory corpuscles. In terms of MFs, they were mainly enriched in calcium release channel activity, electrical conduction activity, and ion channel activity. According to the KEGG enrichment analysis results, these DEGs were mainly enriched in the regulatory pathways related to Parkinson's disease, nonalcoholic fatty liver disease, cell senescence, and bladder cancer (Fig. 4).

### LncRNA expression and functional verification

**Fig. 5:**
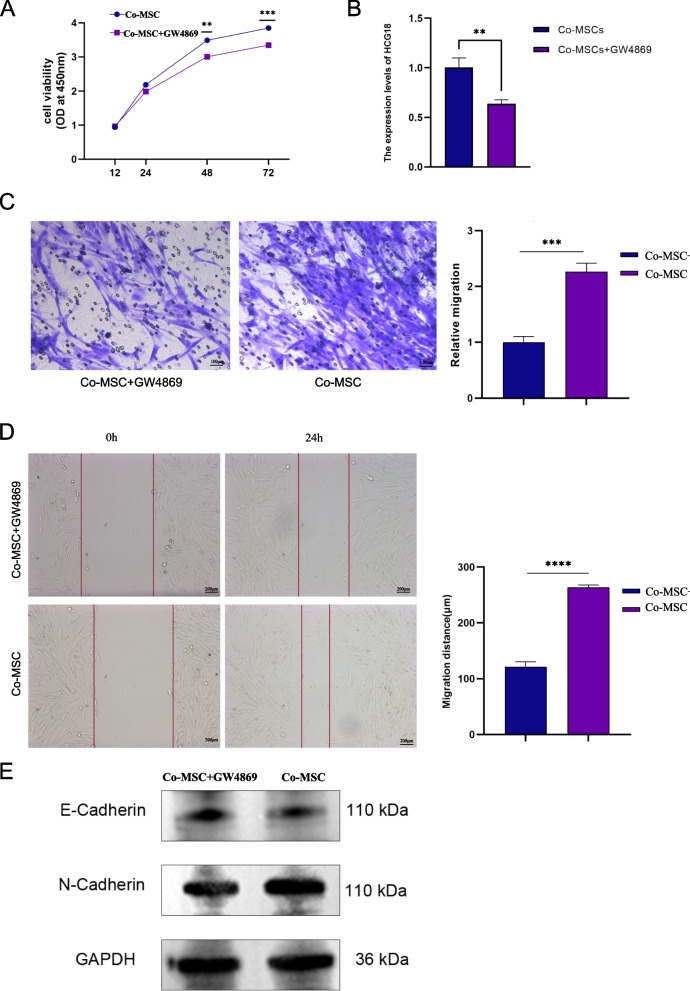
**A** Growth curves of cells in the experimental group and the control group detected by the CCK8 assay. **B** The expression of HCG18 in target cells detected by qRT-PCR. **C** and **D** Detection of cell migration in the experimental group and the control group. **E** The down-regulated expression of N-cadherin and the up-regulated expression of E-cadherin detected by western blotting after the addition of GW4869. *p < 0.05, **p < 0.01, ***p < 0.001

HCG18 is the only lncRNA with high expression in bone marrow-derived MSCs and low expression in hypospadias. Hence, HCG18 was selected as a candidate lncRNA for subsequent experiments. Specifically, human bone marrow-derived MSCs and primary foreskin fibroblasts were co-cultured and then treated with exosome inhibitor GW4869. According to the CCK-8 experiments, the Co-MSCs + GW4869 group had a more significant inhibitory effect on the proliferation of target cells compared to the Co-MSCs group (*P* < 0.05) (Fig. 5A). The synthesis of endogenous HCG18 was inhibited in target cells treated with the transcriptional inhibitor DRB (350 mg/L) for 1 h. RT-qPCR detected that HCG18 was highly expressed in the Co-MSCs group compared to the Co-MSCs + GW4869 group, indirectly proving that HCG18 in the target cells was exosome-derived (Fig. 5B). The results of the Transwell and cell scratch assays suggest that the Co-MSCs + GW4869 group had a more significant inhibitory effect on the migration of target cells compared to the Co-MSCs group (*P* < 0.01) (Fig. 5C, D). Furthermore, Western blotting revealed the expression of EMT-related core proteins following co-culture of MSCs and primary foreskin fibroblasts. In comparison to the Co-MSCs group, the Co-MSCs + GW4869 group exhibited a decrease in the expression of EMT-associated N-cadherin and an increase in the expression of E-cadherin on target cells (Fig. 5E, Supplementary Fig. 2). In addition, we also downregulated the expression of HCG18 by small interfering RNA (siRNA). The results of cellular experiments showed that downregulation of HCG18 expression had a more pronounced inhibitory effect on the proliferation and migration of target cells compared with the si-NC group (*P* < 0.05) (Supplementary Fig. 1). The study showed that exosome-derived HCG18 promotes the occurrence of the EMT process in target cells, as well as the adhesion and migration between epithelial cells. This is expected to facilitate wound healing and urethra repair.

## Discussion

Currently, the incidence of hypospadias is increasing with each passing year, and surgical treatment is an effective option for this disease [20–22]. However, the surgical outcome of hypospadias is still unfavorable, and such complications as severe urinary fistula and urethral stricture cannot be avoided due to poor plastic effects [23, 24]. MSCs have been verified to be useful for carrying therapeutic genes, and these cells present great potential in the field of cell therapy and regenerative medicine [25, 26]. It has been revealed that applying exogenous MSCs contributes to the recovery of many organs and tissues, such as heart, liver, brain, and pancreas. This also provides a new option for eliminating postoperative complications of hypospadias.

In this study, the transcription profiles of genes related to MSC-Exos and hypospadias were collected and analyzed based on GEO. A total of 4 MSC-Exo-derived lncRNAs related to the regulation of EMT in hypospadias were screened. Then, 27 lncRNA-miRNA-mRNA regulatory relationships were obtained by predicting disease-related miRNAs and screening functional lncRNAs. Based on that, a ceRNA regulatory network (including 2 mRNAs, 4 lncRNAs, and 6 miRNAs) was constructed. In recent years, it has been reported that MSC-Exo-derived lncRNAs can affect tissue regeneration and repair through the competitive inhibition of miRNAs. For example, the MSC-Exo-derived lncRNA KLF3-AS1 has been revealed to inhibit the pyroptosis of myocardial cells through the miR-138-5p/Sirt1 axis and promote cell repair, thus reducing myocardial infarction area [27]. In addition, MSCs from gingival tissues can reduce apoptosis through the MEG3/miR-21a-5p/PDCD4 axis, thus protecting the nerve from ischemia–reperfusion injury in the retina [27, 28]. Based on these findings, it can be speculated that the lncRNA-miRNA-mRNA axis in exosomes derived from MSCs may also reduce postoperative complications of hypospadias by promoting the EMT process.

In this study, 4 key lncRNAs were obtained by the microarray data analysis. Among them, HCG18 was the only lncRNA with high expression in bone marrow-derived MSCs and low expression in hypospadias. HLA complex group 18 (HCG18) is a lncRNA. It was first reported to aggravate intervertebral disc degeneration in 2017 [29], and then proved to regulate the proliferation and migration of bladder cancer cells [28]. HCG18 is located on the human chromosome region 4p16.1. There are 4 transcripts of HCG18 in the National Center for Biotechnology Information (NCBI), including NR_0024053.2, NR_102326.1, NR_024052.2, and NR_102327.1. HCG18 has been discussed in myasthenia gravis [30], osteoporosis [31], acute kidney injury after ischemia [32], nonalcoholic fatty liver disease [33], and many other diseases. However, it remains unclear about the specific mechanism of HCG18 in the regulatory network of hypospadias. HCG18 is mainly located in the cytoplasm. Hence, it can be speculated that lncRNA HCG18 may affect the occurrence and development of hypospadias through post-transcriptional ceRNA regulation mechanisms. lncRNAs play an important role in different biological processes through sponge adsorption of miRNAs. For example, exosomes secreted by polymorphonuclear neutrophils may affect the polarization of alveolar macrophages by transferring HCG18 and adsorbing miR-146b, thus resulting in acute pulmonary injury [34]. Exosome-derived lncRNA HCG18 could promote the growth and metastasis of cholangiocarcinoma cells through the miR-424-5p/SOX9 axis of the PI3K/AKT pathway [35]. Exosome-derived lncRNA HCG18 can up-regulate the expression of KLF4 and promote the polarization of M2 macrophages by sponging miR-875-3p in macrophages, thus regulating the progression of gastric cancer [36]. In this study, the results indicated that HCG18 was transported from bone marrow-derived MSCs to target cells through exosomes, and it may promote cell proliferation, migration, and EMT through ceRNA.

## Conclusion

In conclusion, HCG18, an MSC-Exo-derived lncRNA related to the regulation of EMT in hypospadias was screened by bioinformatics analysis. Besides, a lncRNA-miRNA-mRNA network was also constructed. Moreover, it was also demonstrated that HCG18 could be transported to target cells through exosomes, which may promote the proliferation, migration, and EMT of these cells through ceRNA. However, similar to most bioinformatics studies, there are still some limitations in this study. Due to the small sample size in this study, DEGs and related pathways need to be verified by in vitro experiments or other functional studies.

### Supplementary Information

**Supplementary Material 1.**

** Supplementary Material 2.**

** Supplementary Material 3.**

## Data Availability

The datasets generated and analysed during the current study are available in the TARGET database, (https://ocg.cancer.gov/programs/target/data-matrix), andGEO database, (https://www.ncbi.nlm.nih.gov/geo/).
