# Supplementary figures and images for: Identification and analysis of MSC-Exo-derived LncRNAs related to the regulation of EMT in hypospadias
Source: BMC Med Genomics. 2024 Apr 16;17:87. doi: 10.1186/s12920-024-01869-9 (PMC11020336; doi:10.1186/s12920-024-01869-9)

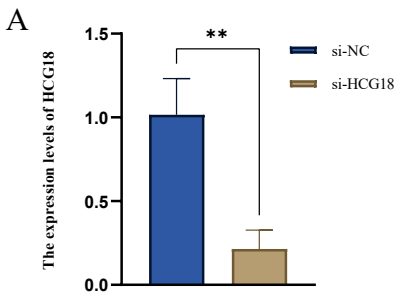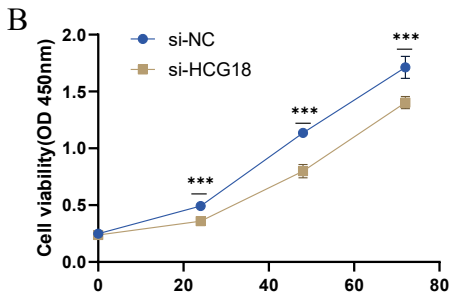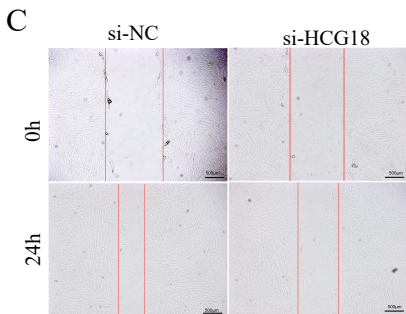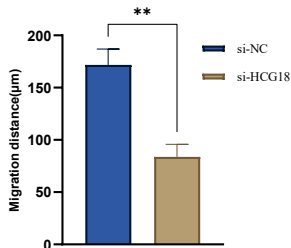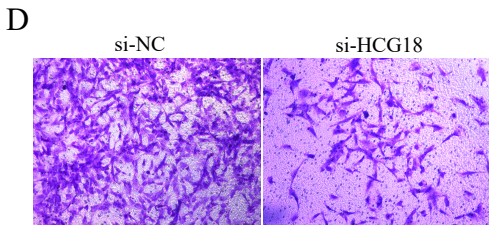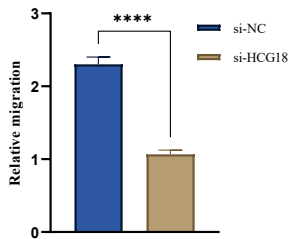

Supplement: Supplementary file 1 — Supplementary Material 1. [file 12920_2024_1869_MOESM1_ESM.pdf]

A

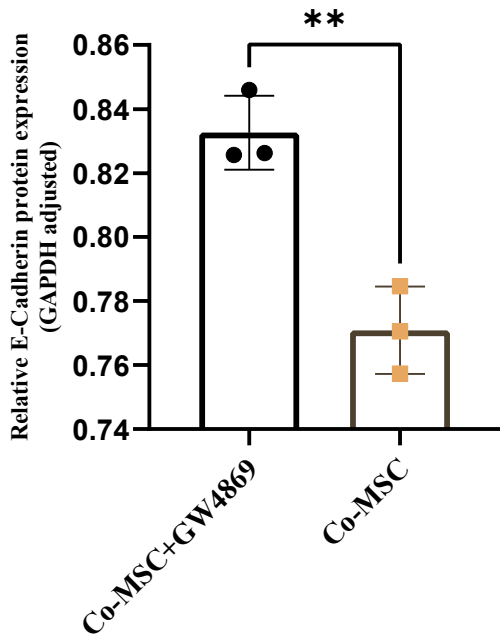

B

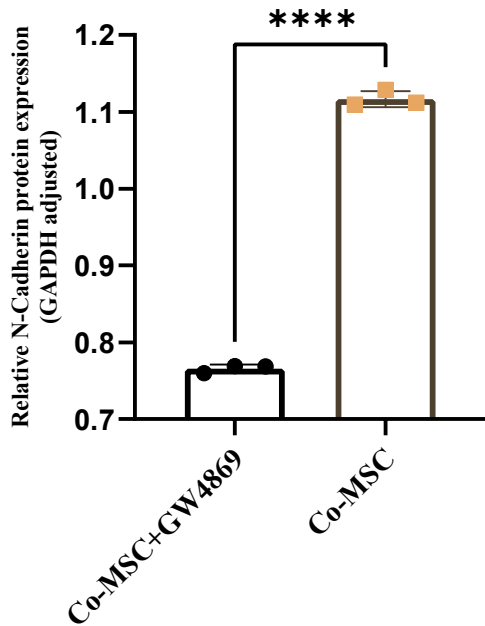

Supplement: Supplementary file 2 — Supplementary Material 2. [file 12920_2024_1869_MOESM2_ESM.pdf]
