# Supplementary material for: Identification and analysis of MSC-Exo-derived LncRNAs related to the regulation of EMT in hypospadias
Source: BMC Med Genomics. 2024 Apr 16;17:87. doi: 10.1186/s12920-024-01869-9 (PMC11020336; doi:10.1186/s12920-024-01869-9)
Supplement: Supplementary file 3 — Supplementary Material 3. [file 12920_2024_1869_MOESM3_ESM.pdf]

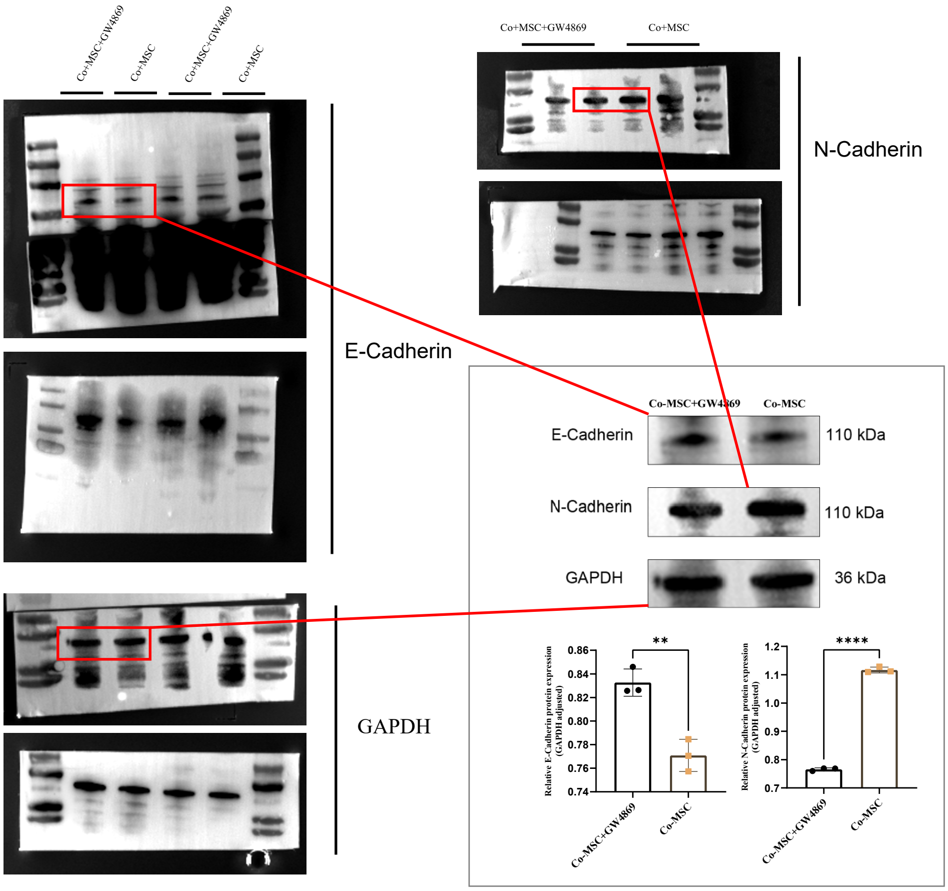

Fig. The relative expression of N/E-Cadherin was detected by Western Blot.
The protein samples were derived from the same experiment.
